# A Case Report on Breast Implant Illness

**DOI:** 10.7759/cureus.45601

**Published:** 2023-09-20

**Authors:** Andrea Asiedu, Qwynton Q Johnson, Sundeep Shah, Akosua S Osafo, Nathan Kumi-Woode

**Affiliations:** 1 Primary Care, Premier Medical Associates, The Villages, USA; 2 Osteopathic Medicine, Kansas City University, Kansas City, USA; 3 Internal Medicine, Premier Medical Associates, The Villages, USA; 4 Medicine, Korle-Bu Teaching Hospital, Accra, GHA

**Keywords:** pleuritic chest pain, breast implant illness, allergy, chronic cough, breast implant

## Abstract

In this case report, we discuss the case of a 64-year-old woman who presented with an unusual complaint of a chronic cough associated with pleuritic chest pain of 15 years following a saline-filled breast implant surgery. Initially, these were minimally abated by acid reflux medications. However, her cough worsened despite other interventions. In the work-up to determine the etiology of her complaints, the most common causes of a chronic cough were considered. The history ruled out post-nasal drip, and pulmonary function tests excluded asthma and chronic obstructive pulmonary disease (COPD), although she had a family history. An IgE allergy panel and an Aspergillus antibody test were also normal. However, an esophagram revealed a significant finding of mild to moderate Gastroesophageal reflux disease (GERD). Ultimately, the subsequent removal of the implants led to a dramatic resolution of her symptoms. It is worth noting that breast implants, like any other medical device, carry certain risks. Complications such as infections, implant rupture, capsular contracture, and changes in breast sensations are known risks associated with breast augmentation surgery.

## Introduction

Breast augmentation has become increasingly popular as more women seek to improve their self-image and address issues such as asymmetry or loss of volume following pregnancy or weight loss. Reports from the American Society of Aesthetic Plastic Surgery provide valuable insight into the procedure's popularity. They reported that approximately 365,000 women had breast augmentation surgeries in 2021 [1]. While the procedure is considered safe, it does carry certain risks and potential complications. These complications include scar tissue formation, which distorts the shape of the breast implant, breast pain, infection, changes in nipple and breast sensation, implant position changes, and implant leakage or rupture. Recent research has raised concerns about the potential association between breast implants and the development of anaplastic large cell lymphoma (ALCL), a rare cancer affecting the immune system. The incidence of ALCL in women with breast implants is, however, extremely low, with an estimated 1-2 cases per million women with implants. Breast implants have been associated with a condition known as "breast implant illness (BII)," which refers to a group of systemic symptoms some women experience after breast augmentation surgery. The exact relationship between breast implants and these symptoms is not well understood, and further research is needed to determine the causes and risk factors for the condition. Reported symptoms of BII can vary, including fatigue, memory loss, skin rashes, concentration problems, and joint pain. These symptoms can be mild to severe and can develop several months or even years after the initial breast augmentation procedure [2]. This case report delves into the rare but significant issue of systemic complications arising from breast implant surgery. The study focuses on a 63-year-old woman who presented with an unusual complaint of a chronic cough and chest pain that had persisted for 15 years following her saline-filled breast implant surgery. Despite ruling out common causes such as post-nasal drip and asthma/chronic obstructive pulmonary disease (COPD), the investigation revealed mild to moderate gastroesophageal reflux disease (GERD) as a potential cause. The report ultimately highlights the importance of considering the potential role of breast implants in developing systemic complications and how removing the implants could lead to dramatic resolution of symptoms.

## Case presentation

**Figure 1:**
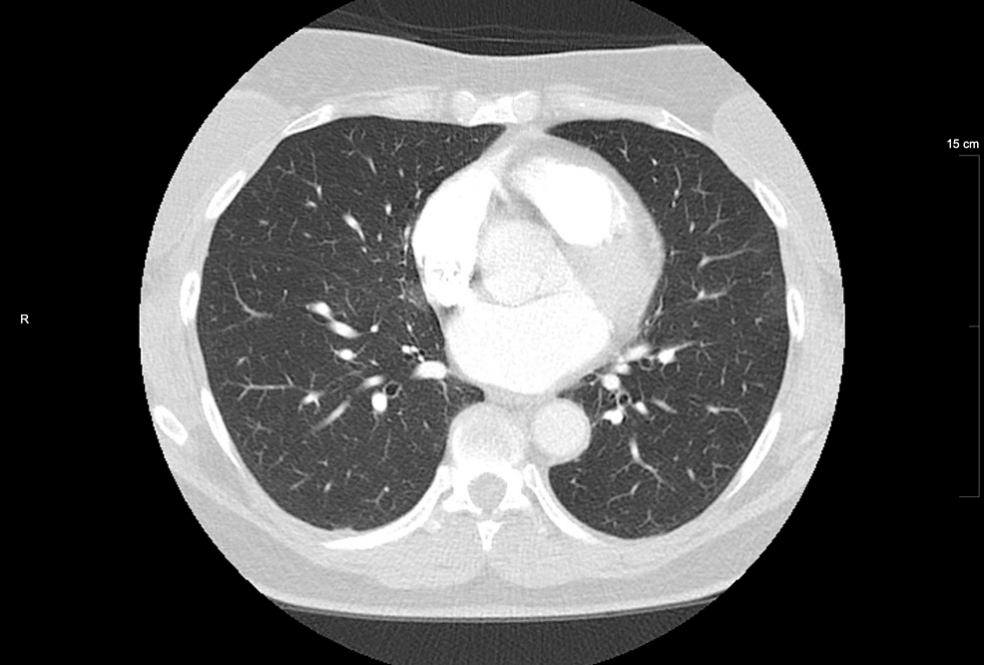
CT angiogram of lung showing atelectasis.

The patient, a 63-year-old, reported for a follow-up and complained of a chronic cough she has had for the past 15 years. In 2020, her cough seemed to worsen despite interventions. Acid reflux medication provided minimal relief previously, but symptoms have exacerbated over the past five years. She described the sensation as being unable to clear her lungs. While the cough was productive, she denied any significant post-nasal drainage. She complained of chest pain during deep breathing and noted that strong smells occasionally triggered the cough. At night, the cough did not disturb her sleep, and she did not experience wheezing. However, she felt short of breath occasionally. The patient has no history of prior pulmonary issues. Notably, her brother has asthma, and her father has COPD. She is a lifelong non-smoker and worked as a social worker in a rehabilitation unit before retiring. She has a dog at home but no known allergies to dogs. It is also worth mentioning that her home does not have carpeting. CT angiogram was negative for pulmonary embolism but showed mild atelectasis, as seen in Figure 1. She had a pulmonary function test (PFT), which was normal. Her six-minute pulmonary stress test was also normal. Overnight oximetry revealed mild hypoxemia, which was not significant. Bronchoscopy findings were normal, but an esophagram revealed a small to moderate GERD. An autoimmune work-up was conducted, with a positive antinuclear antibody (ANA) screen showing a speckled pattern in February 2022 while she was still symptomatic. Both the IgE central allergy panel and the Aspergillus antibody tests were negative.

Finally, a decision to remove the saline breast implants was made in April 2022. Post-op, a fibrous capsule with moderate edema was noted, which was densely adherent to surrounding tissues. Muscle tissue was found to cover approximately 45% of the capsule's surface area, and the capsule was more edematous posteriorly. The nipple-areola complex was shortened due to the tubular deformity. There was adherence to the serratus fascia. The implant examined post-op had clear saline with a small plug of whitish amorphous material floating in each one, consistent with a bit of tissue from the valve guard site. There was moderate yellowing of the envelope. The capsule ranged in thickness from moderately thick areas to thin areas. There was no unusual fluid or calcification. After removal, the patient's cough and chest pain resolved almost immediately, and a repeat ANA screen done six months after breast implant renewal was now negative. 

## Discussion

Breast implant illness

BII is a phenomenon that describes a diverse constellation of symptoms that arise after breast implants. It also represents a controversial and poorly understood aggregation of unclear and always-present complications. They are seemingly idiopathic and can greatly vary. Multiple reports indicate the occurrence of various health issues after patients receive breast implants, although it is essential to note that the evidence on this topic is not yet substantial. These reported illnesses encompass symptoms such as chest pain, anxiety, neurological disorders, the onset of new autoimmune diseases, and the worsening of pre-existing autoimmune conditions. Saline breast implants have been said to be safer than silicone-filled breast implants as the latter is much more foreign to the body and invokes an immune response, as per a study conducted by Kossovsky N and Freiman CJ in 1987 and more recently in 2015 by Nesher G et al. who found four females who had Autoimmune/Inflammatory Syndrome Induced by Adjuvants (ASIA) as a result of rupturing of their silicone breast implant [3,4]. Cosmetically, the appeal of either is arguable, with different studies producing results that suggest that both implant types provide a similar degree of ripple and animation deformity, an undesirable effect of the implant [5]. However, a study by Singh N et al. found no difference in safety in saline versus silicone breast implants. Saline breast implants, as observed in our study involving individuals aged between 16 and 63 years, appear to be just as linked to BII as silicone breast implants. This is supported by our findings, which show that chronic cough symptoms resolved after implant removal [6]. 

A classification system proposed by Cohen Tervaert JW et al. for silicone BII incorporates categorization of the symptoms into five classes: (i) complications that occur locally; (ii) migration of silicone to the skin, lower extremities, and lungs; (iii) surgical side effects such capsular formation and rupture; (iv) allergy to breast implant components; (v) manifestations of systemic inflammation, such as those seen in lymphoma development and autoimmune rheumatic disorders [7].

Relevance of Breast Implant Illness

More research and pursuance of this topic is necessary to allow a more informed decision to be made concerning getting breast implants and explantation in patients with similar symptomatology. Due to the growing interest in the field of cosmetic surgery and specifically breast implants, the need to study the indications for explantation in silicone breast implants because of breast implant illness was studied by Lieffering AS et al. [8]. Their data was obtained from the Dutch breast implant registry. It was a two-fold study: a legacy cohort and a prospective study. The legacy cohort was conducted between April 1, 2015, and December 31, 2020, and a prospective cohort study was conducted between April 1, 2015, and December 31, 2019 (with follow-up until December 31, 2020). Their analysis included 12,882 (6,667 women; mean (SD) age, 50.6 (12.7) years) who had cosmetic breast implants and 2,945 reconstructive breast implants (2,139 women, mean (SD) age, 57.9 (11.3) years) in the legacy study. The study population in the prospective cohort included 47,564 women who had cosmetic breast implants (24,120 women, mean (SD) age, 32.3 (9.7) years) and 5,928 women who had had reconstructive breast implants (4,688 women, mean (SD) age, 50.9 (11.5) years). Their data showed that the median (IQR) time to revision of the implant in the prospective study was 1.8 (0.9-3.1) years in 739 (1.6%) of the women who had cosmetic breast implants and 697 (11.8%) reconstructive breast implants underwent revision after a median (IQR) time to reoperation of 1.1 (0.5-1.9) years. Of the number, 35 (4.7%) cosmetic revisions resulted from BII compared to the five reconstructive revisions, corresponding to 0.1% of the inserted implants.

The legacy study, however, had 536 (4.2%) cosmetic revisions and 80 (2.7%) reconstructive revisions performed because of BII. Their conclusion reflects the infrequency of BII as a reason for an explanation when it applies to silicone breast implants, which is about the same in safety compared to saline breast implants [8].

Existing Literature on the Topic

A study done on breast implant-associated immunological disorders by Suh LJ et al. found an association between breast implants and autoimmune disorders, typified by a very vast combination of symptoms such as fatigue, myalgias/arthralgias, dry eyes/mouth, and rash [9]. Markers for autoimmune diseases such as ANA have also been studied in patients with similar complaints after breast implants. One publication in 2016 looking at the effect of breast implant removal in patients with autoimmune conditions found that although some patients had symptomatic improvement post-explantation, ANA remained positive in all the patients. This contrasts with our patient, who had both symptomatic improvements and negative ANA screen post-explantation [10]. Habib PM et al., in 2022, presented three females who had BII: two with saline breast implants and one with silicone breast implants. For the purposes of our study, only the saline breast implants will be considered. The first was a 49-year-old female who underwent a saline breast augmentation in May 1994 for cosmetic purposes. The patient presented three years later with a major complaint of non-specific chest pain and other symptoms of fatigue, tinnitus, and daily headache, amongst others. Her laboratory test results and imaging work-up were unremarkable, mirroring those of the patients in our study. However, her past medical history was significant for untreated rheumatoid arthritis, hypothyroidism, and premature ventricular contractions. She had no known drug allergies and an unremarkable family and social history. She had an explanation due to the BII and experienced a resolution of symptoms after a week despite no changes in her prior medications [11].

The second was a 50-year-old who had her saline implant in 2003 for cosmesis and, after two years, began to experience recurrent respiratory infections among other non-specific symptoms, most notably, vision changes. She had Sjogren's syndrome six months after the implant. Her past medical history included anxiety, chronic yeast infection, and antithrombin 3 deficiency. Her social history was significant for smoking half a pack a day. She had the implants removed and had an improvement in her condition one week post-operatively. A month later, she had significant resolution and returned to near-normal function. The past medical, family, and social history in both cases were inconsequential to the resolution of their symptoms as both report relief of symptoms post-explantation, just as in the case of our study [11].

­­*Comparison With Similar Presentations*

Considering the duration of her symptoms and their resolution after the explantation, we have effectively ruled out infectious causes of chronic cough. Additionally, the negative results from imaging studies have allowed us to dismiss malignancy and foreign bodies in the airway as potential explanations for her symptoms.

COPD: Chronic respiratory symptoms (dyspnea, coughing, and sputum production) caused by abnormalities of the airways (bronchitis, bronchiolitis) and/or alveoli (emphysema), which result in persistent, frequently progressive airflow obstruction, characterize COPD, a heterogeneous lung condition [12]. Chronic cough is a usual symptom and almost always the first presentation of the disease, and it can either be productive (30% of cases) or non-productive. Our patient developed a productive cough, still within the expanse of COPD. She also had a positive family history of the disease, notably that her father had the disease. She was a non-smoker, another significant risk factor for developing the disease. The gold standard for diagnosis of the disease is bronchodilator spirometry. Her PFT was unremarkable, as well as her six-minute test. These are completely unusual in the case of COPD. The damage sustained by the lungs is usually irreversible in COPD. We found that the patient's symptoms completely resolved post-removal of the implants.

GERD: BII, in this particular case, initially resembled GERD. Although GERD more commonly presents with heartburn, multiple reports have documented unusual symptoms, including non-cardiac chest pain, laryngitis, asthma, and chronic cough [13].

In our study, for the patient who presented with a chronic cough and chest pain, GERD was initially considered a differential diagnosis, especially given that an esophagram revealed small to moderate GERD. In a prospective study, about 21% of 102 immunocompetent patients with GERD experienced a chronic cough over a 22-month interval [14].

The cough associated with GERD is typically longstanding, worse during the day, and more pronounced in the upright position. Although the patient did not exhibit the classic symptom of heartburn, reported cases of atypical GERD have shown that more than half of patients with a chronic cough do not simultaneously experience heartburn. This has led many cases of atypical GERD to be misdiagnosed. Adding to the challenge is the fact that upper GI endoscopy and 24-hour pH monitoring may not always be sensitive enough to diagnose atypical GERD.

The pathophysiological pathways for these unusual symptoms, such as chronic cough and asthma, include micro-aspiration of gastric contents and vagally mediated stimulation of acid-sensitive receptors in the respiratory tract due to increased acidity in the lower esophagus. Furthermore, due to the common embryologic origin of the esophagus and the bronchial tree, acid-sensitive receptors in the bronchial tree can also be stimulated, leading to pulmonary symptoms of GERD, including chronic cough. Gastric acid suppression is the most commonly employed approach for patients with suspected GERD presenting with unusual symptoms. In this study, the patient experienced mild relief followed by an exacerbation of symptoms after acid suppression therapy, making GERD an unlikely etiological factor [12].

Invasive Aspergillosis: It often presents in immunocompromised patients and is a major cause of morbidity and mortality in these patients. However, pulmonary aspergillosis can also occur in patients with an intact immune system. This usually occurs after massive fungal spore exposure. It is also important to note that chronic pulmonary aspergillosis is much more likely to occur in immunocompetent patients when there is concomitant lung pathology such as COPD, sarcoidosis, or tuberculosis (TB), none of which the patient in the case report had.

Allergic bronchopulmonary aspergillosis (ABPA): It presents with persistent respiratory symptoms, including a chronic cough, which is common in patients with asthma and cystic fibrosis. It presents in a few cases as persistently uncontrolled asthma, which is unresponsive to the intensive treatment for asthma. This condition, when left untreated, may lead to permanent airway damage. In this patient with a chronic cough for the past 15 years, it is essential to explore the possibility of atypical aspergillosis. IgE panel and aspergillus antibody were, however, normal. A high-resolution computed tomography (HRCT) scan of the thorax would have typically revealed bilateral reticulonodular lesions or airway damage in patients with the condition. For our patient, it only picked up mild atelectasis. These findings, combined with a negative past medical history, made ABPA unlikely [15].

## Conclusions

We brought to light in this case report the unlikely cause of a chronic cough that lasted for over 15 years: a saline breast implant. Though saline breast implants are generally considered safe, it is essential to acknowledge that they can still be associated with the constellation of symptoms referred to as BII. While the symptoms and complications of BII vary and are poorly understood, their resolution occurred almost immediately after removing the implants in this patient.
